# MMP-14 (MT1-MMP) Is a Biomarker of Surgical Outcome and a Potential Mediator of Hearing Loss in Patients With Vestibular Schwannomas

**DOI:** 10.3389/fncel.2020.00191

**Published:** 2020-07-28

**Authors:** Yin Ren, Hiroshi Hyakusoku, Jessica E. Sagers, Lukas D. Landegger, D. Bradley Welling, Konstantina M. Stankovic

**Affiliations:** ^1^Eaton Peabody Laboratories, Department of Otolaryngology—Head and Neck Surgery, Massachusetts Eye and Ear, Boston, MA, United States; ^2^Department of Otolaryngology—Head and Neck Surgery, Harvard Medical School, Boston, MA, United States; ^3^Division of Otolaryngology—Head and Neck Surgery, University of California, San Diego, San Diego, CA, United States; ^4^Department of Otorhinolaryngology, Yokosuka Kyosai Hospital, Kanagawa, Japan; ^5^Program in Speech and Hearing Bioscience and Technology, Harvard Medical School, Boston, MA, United States; ^6^Harvard Program in Therapeutic Science, Harvard University, Boston, MA, United States

**Keywords:** MMP-14, vestibular schwannoma, biomarker, hearing loss, surgical outcome

## Abstract

Improved biomarkers are needed for vestibular schwannoma (VS), the most common tumor of the cerebellopontine angle, as existing clinical biomarkers have poor predictive value. Factors such as tumor size or growth rate do not shed light on the pathophysiology of associated sensorineural hearing loss (SNHL) and suffer from low specificity and sensitivity, whereas histological markers only sample a fraction of the tumor and are difficult to ascertain before tumor treatment or surgical intervention. Proteases play diverse and critical roles in tumorigenesis and could be leveraged as a new class of VS biomarkers. Using a combination of *in silico*, *in vitro*, and *ex vivo* approaches, we identified matrixmetalloprotease 14 (MMP-14; also known as MT1-MMP), from a panel of candidate proteases that were differentially expressed through the largest meta-analysis of human VS transcriptomes. The abundance and proteolytic activity of MMP-14 in the plasma and tumor secretions from VS patients correlated with clinical parameters and the degree of SNHL. Further, MMP-14 plasma levels correlated with surgical outcomes such as the extent of resection. Finally, the application of MMP-14 at physiologic concentrations to cochlear explant cultures led to damage to spiral ganglion neuronal fibers and synapses, thereby providing mechanistic insight into VS-associated SNHL. Taken together, MMP-14 represents a novel molecular biomarker that merits further validation in both diagnostic and prognostic applications for VS.

## Introduction

Vestibular schwannoma (VS) is the fourth most common intracranial tumor and constitutes 90% of all cerebellopontine angle (CPA) lesions (Mahaley et al., 1990). It is a benign, slow-growing tumor arising from neoplastic Schwann cells lining the eighth cranial nerve. While the majority of VS occurs sporadically, a portion develops in association with neurofibromatosis type II (NF2). Despite its non-malignant nature, growing VS can be associated with significant morbidity including sensorineural hearing loss (SNHL) that affects up to 95% of patients, tinnitus, dizziness, increased rates of hospitalization, and poor mental health (Genther et al., 2013). As tumors grow larger, they can cause additional symptoms such as palsy of the facial nerve and other cranial nerves, and potentially life-threatening complications including brainstem compression. The goal of contemporary VS management is to balance long-term tumor control, neurological function, and quality of life (Carlson et al., 2015b). The standard treatment to remove the tumor is limited to surgery, as there are currently no FDA-approved pharmacotherapies for the treatment of VS or associated SNHL. Nonetheless, surgery can be associated with significant morbidities such as facial paralysis and deafness.

While numerous retrospective studies have attempted to identify clinical factors associated with disease severity and prognosis, there are no established reliable biomarkers to predict the degree of VS-associated SNHL or surgical outcomes after tumor resection. Clinical factors such as tumor size, tumor growth rate, and radiographic imaging features often suffer from poor sensitivity and specificity. Histological findings such as tumor proliferative index, while useful, are obtained only after tumor extirpation and do not offer prognostic information (Niemczyk et al., 2000). Moreover, there is great variability in surgical outcomes when patients undergo attempted microsurgical tumor resection. Factors such as tumor consistency, presence of cystic changes, and adhesions between the tumor capsule and the facial nerve have all been associated with an increased likelihood of subtotal tumor resection (STR; Moon et al., 2007). There have been over twenty clinical trials in the last 20 years on experimental therapies for NF1 and NF2, yet no biomarker-driven trial for NF2 has been performed (Plotkin et al., 2013; Hanemann et al., 2016). Further, little is known about the molecular underpinnings of VS tumorigenesis that predisposes certain tumors towards incomplete resections.

Molecular biomarkers are objectively measured indicators of disease pathophysiology and have the potential to facilitate early detection of tumors, monitor disease progression, and impact the outcomes of treatment. Proteases, a class of enzymes that plays a fundamental role in both normal tissue remodeling and aberrant cellular growth, are candidates for biomarkers for VS (Kessenbrock et al., 2010). In many human cancers, protease biomarkers have been well established to assess treatment response and even predict survival (Michael et al., 1999; Ricci et al., 2017). Various members of the matrix metalloprotease (MMP) family have been implicated in promoting VS growth *via* angiogenesis, remodeling of the extracellular matrix, or enhancing growth factor signaling pathways (Cayé-Thomasen et al., 2003; Moon et al., 2007; Møller et al., 2010). While these results are promising, the studies are limited to a small number of patients, are retrospective, and address only a particular protease candidate. Additionally, the majority of the published studies rely on immunohistochemical staining of tumor specimens rather than measuring serum levels as a readout, which precludes preoperative patient counseling and forecasting of clinical outcomes.

Since hearing loss is the most common consequence of VS, a clinically useful biomarker should differentiate tumors with good hearing from those with poor hearing. Furthermore, given the variability in clinical outcomes such as the extent of surgical resection, the biomarker should provide information about the likelihood of gross total tumor removal and the functional outcome of the facial nerve; two features that would greatly impact both long-term prognosis and the patient’s quality of life. Finally, a protease-based biomarker has the potential to outperform existing biomarkers which are often disease by-products with insufficient sensitivity, through leveraging the catalytic nature of protease signal amplification.

To this end, we utilized transcriptomic data from the largest analysis to date of patients with VS to identify, for the first time, several proteases aberrantly expressed in VS genomes. Matrix metalloprotease 14 (MMP-14, also known as membrane type 1 MMP, MT1-MMP) was selected for further analysis and was found to have elevated expression and activity in an NF2-derived cell line, primary human VS cultures, and plasma samples from patients with VS. We ascertained the usefulness of MMP-14 as a potential biomarker by identifying its association with surgical outcomes. Finally, the mechanism by which MMP-14 could cause VS-associated SNHL was investigated in cochlear explant models.

## Materials and Methods

### Study Population and Human Specimen Collection

Freshly harvested human VS tumor specimens from patients with sporadic VS and control great auricular nerve (GAN) were collected from indicated surgeries and transported to the laboratory in saline on ice, as is routine in our laboratory (Dilwali et al., 2015b). From July 2015 to June 2018, blood samples were collected prospectively from patients undergoing surgical resection on the day of surgery, typically with 30 min of inducing general anesthesia, and at least an hour before starting tumor microdissection. Informed consent was obtained from all patients. Fresh blood was collected in plasma preparation tubes (Beckton Dickinson, NY, USA). The blood was spun at 2,000 g for 10 min at 4°C. Plasma was separated and spun at 2,000 g for 5 min at 4°C. Centrifuged plasma was filtered through 0.8 μm filters and stored at −80°C until further use. VS secretions were collected as previously described (Dilwali et al., 2015b; Landegger et al., 2017). Briefly, VS specimens were rinsed with sterile PBS thrice, then incubated in 100% DMEM for 3 days at 37°C and 5% CO_2_ levels in sterile conditions. Tissue secretions (i.e., tissue-conditioned media) were normalized by weight (0.1 g specimen/0.1 ml DMEM). Control DMEM without tissue was incubated in parallel in a separate tube. VS secretion was collected after removing the tissue specimen and frozen at −80°C until further use.

All study protocols were approved by the Human Studies Committee of Massachusetts General Hospital and Massachusetts Eye and Ear and conducted following the Helsinki Declaration. The human VS cell line HEI-193, derived from a patient with neurofibromatosis type 2 (NF2), was obtained from Dr. Giovannini’s team at the University of California, Los Angeles.

### Clinical Data Collection

Clinical charts, operative reports, pre- and post-operative radiographic imaging, and pathology reports were reviewed. The degree of tumor resection was determined based on operative descriptions as well as postoperative contrast-enhanced MRI scans. Gross tumor resection was defined as complete tumor removal; near-total resection was defined when a small tumor remnant, no greater than 5 × 5 × 2 mm, was left behind to preserve nerve continuity. In all other scenarios, a STR was assigned. Patients who underwent either gross-total or near-total resections were classified together as one cohort (GTR) whereas those with sub-totally resected tumors were classified as a second cohort (STR). All tumors were removed *via* either a retrosigmoid craniotomy approach (RS; 18 out of 23) or a translabyrinthine approach (TL; five out of 23). Facial nerve function was described using the House-Brackmann (HB) grading scale. For annotation of the patient data, variables were defined using previously established criteria. These included: age (defined at the time of diagnosis), tumor volume, pure tone average (PTA; the four-point average measured at 0.5, 1, 2 and 3 kHz) and word recognition (WR; the percentage of spoken monosyllabic words a subject can discern from a list typically read at 70 dB or at the level at which a patient’s speech intelligibility curve plateaus). Audiometric data were obtained from the latest measurements before tumor resection surgery. A PTA of 100 dB and WR score of 0% were assigned to a deaf ear. All patients underwent gadolinium-enhanced magnetic resonance imaging (MRI). Tumor volume was measured on axial contrast-enhanced T1-weighted MRI using a Voxar unit (Toshiba Medical Visualization Systems, Edinburgh, UK) as previously described (Kandathil et al., 2016). Tumor volumes were independently measured, averaged, and expressed in milliliters (cubic centimeters). Tumor volume measurements were performed on MRI scans closest to the time of and typically within 3 months of surgical resection. Growth rates were determined by comparing tumor volumes between the last MRI before surgery and the first MRI at the time of tumor diagnosis when available; these scans were typically at least 6 months apart.

### Ingenuity Pathway Analysis

Ingenuity Pathway Analysis (IPA; Qiagen Bioinformatics, Redwood City, CA, USA) was used to perform standard Core Analysis on the genes in the meta-analysis that reached significance after Bonferroni correction for multiple hypotheses testing (*P* < 0.05). Statistical analysis of IPA data was performed using the right-tailed Fisher’s exact test.

### Cell Culture

Details on primary VS and human Schwann cell cultures have been described previously (Landegger et al., 2017). Briefly, freshly harvested VS or GAN specimen was rinsed in phosphate-buffered saline (PBS), dissected and maintained in culture medium consisting of Dulbecco’s Modified Eagle’s medium (DMEM) with Hams’ F12 mixture, 10% fetal bovine serum (FBS), 1% Penicillin/Streptomycin (Pen/Strep) and 1% Glutamax (Life Technologies, Carlsbad, CA, USA). GAN samples were used as controls because: (1) like the cochleovestibular nerve, GAN is a sensory nerve with a robust Schwann cell layer; and (2) healthy GANs are readily accessible from routine neck dissection surgeries. The cochlear nerve was not selected as the control because it travels close to the vestibular nerve at the root entry zone, is located adjacent to and may be adherent to the tumor capsule, and may harbor molecular changes from the surrounding tumor microenvironment. The primary VS and GAN cultures were maintained for 2–4 weeks as described previously (Landegger et al., 2017). The human schwannoma cell line HEI-193 was cultured in DMEM with 10% FBS, 2 mM glutamine, and 1% Pen/Strep.

### Quantitative Assay of MMP Activity

Fluorogenic peptide substrates (Enzo Life Sciences, Farmingdale, NY, USA and CalBioChem, San Diego, CA, USA) sensitive to cleavage by proteases including MMP-14 were incubated with either patient plasma or tumor secretions. The fluorescence signal was measured at specified time points. For inhibition experiments, MMP inhibitors were added before incubation with peptide substrates. Specifically, fluorogenic peptides were incubated with samples for 30 min at 25°C before detection. The final volume was 100 μl in MMP-specific buffer, consisting of 50 mM Tris, 150 μM NaCl, 5 μM CaCl_2_, 1 μM ZnCl_2_, pH 7.5. Fluorescence excitation wavelength at 328 nm with emission at 400 nm was measured in an automated fluorescent plate reader at 37°C (SpetroMax Gemini EM Microplate Reader, Tecan Life Sciences, Mannedorf, Switzerland). Both plasma and tumor secretion samples were diluted 1:20 to ensure that signal levels were not saturated. Michaelis-Menten constants were calculated based on initial substrate cleavage velocities at varying substrate concentrations (GraphPad Prism 5.0, San Diego, CA, USA). The broad-spectrum MMP inhibitor Marimastat (Millipore Sigma, Danvers, MA, USA) and MMP-14 specific inhibitor NSC405020 (Tocris Bioscience, Minneapolis, MN, USA) were added at a final concentration of 100 μM. The level of MMP-14 in tumor-conditioned media, GAN-conditioned media and peripheral blood were determined by ELISA per the manufacturer’s protocol (Abcam, Cambridge, MA, USA).

### Immunofluorescence and Immunohistochemical Staining of Tumor Sections

Immunohistochemical staining was performed using streptavidin-biotin 3-step indirect methods. Specifically, formaldehyde-fixed paraffin-embedded (FFPE) tumor tissues were sectioned, deparaffinized with xylene, underwent antigen-retrieval in citrate buffer, and stained using polyclonal antibodies against MMP-14 (1:1,500, Abcam, Cambridge, MA, USA). Endogenous peroxidase activity was blocked with hydrogen peroxide, and the slides were blocked with 5% bovine serum albumin in Tris-buffered saline. Sections were incubated with primary antibodies, washed thoroughly, then incubated with peroxidase-labeled polymer and developed using the DAB HRP substrate. Slides were counterstained with hematoxylin. For tumor immunofluorescence, after incubation with primary antibodies overnight at 4°C, the slides were thoroughly washed and incubated with corresponding anti-rabbit secondary antibodies for 1 h at room temperature, and cell nuclei were counterstained with DAPI stain (Invitrogen, Carlsbad, CA, USA). The tissue slides were imaged using a Carl Zeiss 2000 upright microscope. The extent and intensity of MMP-14 expression were semi-quantitatively evaluated in a blinded fashion. Scoring was classified into the following four groups based on a combination of the staining intensity and the percent of positively stained cells: 0+, no expression (weak staining intensity and ≤10% positive cells); 1+, low expression (weak staining intensity, greater than 10% but ≤25% positive cells); 2+, moderate expression (moderate staining intensity, greater than 25% but ≤50% positive cells); and 3+, high expression (moderate to intense staining intensity, greater than 50% positive cells). For each tumor specimen, at least 8 to 10 randomly selected histological sections were chosen for quantification. For GAN staining, seven (Plotkin et al., 2013) different specimens from unique patients were used.

### Quantitative RT-PCR

Reverse transcriptase (RT)-PCR was performed using Applied Biosystems Step One Plus Real-time PCR. Specifically, to measure the expression levels of members of the MMP family and GAPDH, total RNA was extracted from tumors using RNA isolation kit and purified using RNeasy columns (Qiagen, Germantown, MD, USA). The RNA templates were used in reverse transcriptase (RT)-PCR reactions using random hexamer primer cocktails and SuperScript III reverse transcriptase (Invitrogen, Carlsbad, CA, USA). Quantitative real-time PCR was performed with an instrument using the SYBR Green PCR Master Mix. Each 25 μl PCR reaction consisted of 12.5 μl 2X SYBR Green qPCR Master Mix (Applied Biosystems, Foster City, CA, USA), 10.5 μl of ddH_2_O, 1.0 μl of cDNA template containing up to 100 ng of cDNA, and 1.0 μl of paired PCR primers. RT-PCR was carried out as follows: Cycle 1: 95 °C for 10 min; Cycle 2: 40 cycles of 95°C for 10 s and 60°C for 60 s. The primer sequences were: MMP-2 Forward: CCCCAAAACGGACAAAGAG, MMP-2 Reverse: CACGAGCAAAGGCATCATCC; MMP-7 Forward: GGTCACCTACAGGATCGTATCATAT, MMP-7 Reverse: CATCACTGCATTAGGATCAGAGGAA; MMP-9 Forward: CACTGTCCACCCCTCAGAGC, MMP-9 Reverse: GCCACTTGTCGGCGATAAGG; MMP-14 Forward: CGCTACGCCATCCAGGGTCTCAAA, MMP-14 Reverse: CGGTCATCATCGGGCAGCACAAAA; MMP-17 Forward: GACCTGTTTGCAGTGGCTGT, MMP-17 Reverse: ACGATCTTGTGGTCGCTGGT; MMP-19 Forward: CAGGCTCTCTATGGCAAGAA, MMP-19 Reverse: GAGCTGCATCCAGGTTAGGT; GAPDH; GAPDH Forward: GGATTTGGTCGTATTGGG, GAPDH Reverse: GGAAGATGGTGATGGGATT.

Triplicate reactions for the gene of interest and the endogenous control were performed separately on the same cDNA samples, and five distinct tumor specimens were used for each MMP gene. For data analysis, the melting curves were verified and only curves with one melting peak were used. GAPDH housekeeping gene was used as the endogenous control. C_T_ values were geometrically averaged and used for ΔΔC_T_ calculations. The relative change in gene expression was calculated as 2^−Δ(ΔCT)^.

### Cochlear Explant Culture and Immunofluorescence Imaging

Murine cochlear explant cultures were established according to previously published protocols (Dilwali et al., 2015b). Briefly, temporal bones from CBA/CaJ mice (Jackson Laboratory, ME, USA) were separated from the skull on postnatal day 4 and the bony otic capsule was removed. The spiral ligament was stripped away starting from the base of the cochlea. The lower apical and upper basal turns were separated into two parts while preserving the sensory organ including the hair cells and spiral ganglion neurons (SGNs). After removal of Reissner’s membrane, the explants were plated onto 10 mm glass coverslips coated with Cell-Tak (BD Biosciences, East Rutherford, NJ, USA) in culture medium consisting of 97% DMEM, 1% FBS, 1% ampicillin and 1% N2 supplement at 37°C in 5% CO_2_. After at least 12 h in culture and confirming the explants have successfully attached to the coverslips, the cultures were then treated with murine MMP-14 (Biomart, Shirley, NY, USA) in DMEM at various concentrations for 72 h. Explants from age-matched littermates receiving no treatment served as negative controls and were analyzed simultaneously using immunofluorescence.

For immunofluorescence imaging of cochlear explants, the following antibodies were used: myosin 7a to label hair cells (Myo7A 1:500, Proteus Biosciences, CA, USA), neurofilament H to label SGN fibers (NF-H 1:2,500, Millipore Sigma, MA, USA), CtBP2 (1:1,000, BD Biosciences, East Rutherford, NJ, USA) to label presynaptic ribbons, PSD95 (1:1,000, Neuromab, Davis, CA, USA) to label postsynaptic ribbons. Explants were washed in PBS, mounted, and incubated with corresponding fluorescent secondary antibodies according to previously published protocols (Dilwali et al., 2015b; Kujawa and Liberman, 2015).

Explants were imaged with a Leica TCS SP8 confocal microscope (Leica Biosystems, IL, USA). Zoomed-in images focusing on the organ of Corti were merged into z-stacks and the number of inner hair cells (IHCs), outer hair cells (OHCs), SGNs, and juxtapositions between CtBP2-positive presynaptic ribbons and PSD95-positive postsynaptic densities were manually counted per 100 μm length. The SGN fibers were analyzed approximately 10 μm beneath the basolateral pole of IHCs. All image analyses were performed by a researcher (HH) blinded to the experimental condition.

### Statistical Analyses

Analysis of genome-wide transcriptomic data of VS and control nerves has been described in detail elsewhere (Sagers et al., 2018). Briefly, two genomic datasets were used in the meta-analysis: an NCBI GEO dataset (GSE39645), which contained 28 sporadic VS, three NF2-associated VS, and eight control nerves (Torres-Martín et al., 2013); and a dataset containing 36 sporadic VS, 13 NF2-associated VS, and seven control nerves (Agnihotri et al., 2014). The data from GSE39645 was analyzed using GEO2R, which provided a list of probes ranked according to differential expression calculated using the *limma* package in R (Ritchie et al., 2015) as well as *p*-values and t-statistics. The Agnihotri dataset was annotated and normalized using the *justRMA* function in the *affy* package in R (Gautier et al., 2004). Following this, each dataset was consolidated to gene-level differential expression and t-statistics were converted to Cohen’s *d* statistics and standard error values, and resulting values were combined by gene using a fixed-effects meta-analysis using the *rmeta* package in R (Lumley, 2018). Genes with significantly differential expressions were selected based on a Bonferroni-corrected *p*-value of less than 0.05 as the threshold of significance. Next, data from GSE39645 and Agnihotri et al. (2014) were combined in a meta-analysis by first removing genes that were not measured in both sets, then combining Cohen’s *d* and standard error values using a fixed-effects meta-analysis (using *meta.summaries* from the *rmeta* package in R).

Continuous factors were summarized with means and ranges, categorical features were summarized with frequency counts and percentages. A comparison of factors between GTR and STR cohorts was evaluated using the Wilcoxon rank-sum and Chi-square tests. Tukey’s *post hoc* multiple comparison tests were performed in ANOVA analysis (GraphPad Prism 5.0, San Diego, CA, USA). Two-tailed Student’s *t*-test was used for comparisons. Statistical analyses were performed using SPSS (IBM, Armonk, NY, USA) and *P*-values < 0.05 were considered statistically significant.

## Results

### Patient Demographics

Primary VS cultures were derived from tumors resected from patients with unilateral sporadic VSs *via* either retrosigmoid (RS) or translabyrinthine (TL) craniotomy approaches. GAN specimens were obtained from surgically sacrificed nerves during neck dissections or parotidectomies as negative controls, both per established protocols (Dilwali et al., 2014). Clinical characteristics from patients are summarized in Table 1. RS or TL craniotomy approach did not confound whether there was gross- or near-total (GTR) or subtotal resection (STR) of the tumor (*P* = 0.86).

**Table 1:** Demographics of patients in the study.

ID	Age (years)	M/F	Tumor volume (ml )	Ipsilateral ear		Contralateral ear		Surgical outcome	Approach
				PTA (dB)	WRS (%)	PTA (dB)	WRS (%)		
1	33	F	35.0	100	0	N/A	N/A	STR	RS
2	54	M	2.5	8	100	18	94	GTR	RS
3	41	M	9.4	18	98	5	100	GTR	RS
4	46	F	45.3	100	0	15	76	STR	RS
5	61	F	5.5	65	4	10	100	STR	TL
6	73	M	7.1	100	0	12	92	GTR	TL
7	56	M	16.2	10	82	10	100	GTR	RS
8	71	M	8.5	65	0	10	94	GTR	RS
9	18	F	5.7	100	0	2	98	STR	RS
10	57	F	24.4	53	8	3	100	STR	RS
11	57	M	23.4	100	0	5	100	STR	RS
12	50	M	3.9	35	22	10	100	GTR	RS
13	60	M	4.6	45	54	20	92	GTR	RS
14	68	M	12.7	100	0	11	98	STR	RS
15	40	M	13.6	50	18	13	94	GTR	RS
16	20	F	3.6	100	0	11	100	STR	TL
17	29	M	2.8	40	48	20	98	GTR	RS
18	68	M	10.2	100	54	85	2	STR	RS
19	70	F	7.0	100	0	10	96	STR	TL
20	49	M	8.8	100	0	25	95	STR	RS
21	65	F	0.5	28	96	15	96	GTR	RS
22	30	F	2.1	80	0	8	92	GTR	TL
23	36	F	7.8	75	0	15	92	GTR	RS

*Tumor ID, subject age, gender, tumor volume (ml), pure tone average (PTA, dB), word recognition score (WRS, %) for the ipsilateral and contralateral ears, surgical outcome (STR, subtotal resection; GTR, gross total resection), and surgical approach (RS, retrosigmoid; TL, translabyrinthine) are shown. PTA is calculated using the four-point average at 0.5, 1, 2 and 3 kHz. N/A, not available*.

### Identification of Differentially Expressed Proteases in VS

**Figure 1:**
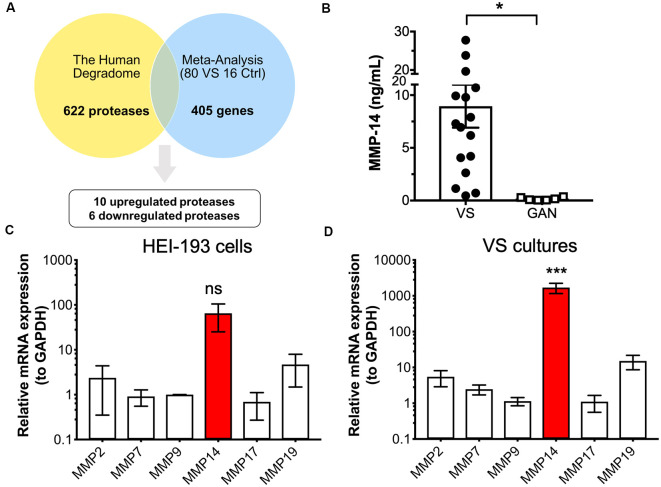
Identification of protease candidates differentially expressed in vestibular schwannoma (VS). **(A)** Schematic of workflow utilizing the Human Degradome and meta-analysis of genome-wide expression in VS, yielding 16 differentially expressed proteases with 10 being significantly upregulated after Bonferroni correction (*P* < 0.05). **(B)** Matrixmetalloprotease 14 (MMP-14) levels are significantly elevated in VS secretions (*n* = 16) when compared to great auricular nerve (GAN) controls (*n* = 6). The mean ± SEM is denoted by a bar and error bars; two-tailed Student’s *t*-test, *P < 0.05. **(C)** Gene expression of several members of the MMP family in HEI-193 human schwannoma cell line as determined by qRT-PCR. *N* = 4 independent experiments. Error bars represent SEM, ns, not significant by one-way ANOVA with Tukey’s *post hoc* multiple comparison tests between MMP-14 gene expression and expression of other proteases. **(D)** MMP gene expression in primary VS cultures (*n* = 5). Error bars represent SEM, ***P < 0.001 for all comparisons between the expression of and other proteases, by one-way ANOVA with Tukey’s *post hoc* multiple comparison tests.

The first objective was to identify protease enzymes that are differentially expressed in VS compared to normal tissue counterparts. Our laboratory reported the largest meta-analysis to date of primary VS tissue, which comprises genome-wide transcriptomic data from 80 VS tumors and 16 control nerve samples (Torres-Martín et al., 2013; Agnihotri et al., 2014). Combined analyses identified a total of 405 genes that are significantly dysregulated in VS (Sagers et al., 2018). We focused on selecting aberrantly expressed protease enzymes using several different methodologies. First, we cross-referenced this set of dysregulated genes with the human Degradome, a collection of 622 human protease genes as well as their inhibitors and substrates (López-Otín and Overall, 2002; Figure 1A). To narrow the focus on protease candidates that have been implicated in tumor progression, tissue invasion, and inflammation, we also compared this gene set with a curated list of cancer proteases from the literature which included MMPs, blood-borne proteases, and families of a disintegrin and metalloproteinase (ADAM) and a disintegrin and metalloproteinase with thrombospondin motifs (ADAMT; López-Otín and Bond, 2008; Supplementary Figure S1A). These two approaches yielded 16 proteases, ten of which were significantly upregulated in VS, and six were significantly downregulated (Supplementary Table S1). Specifically, genes encoding protease enzymes with an upregulated expression included members of the serine protease family (*PRSS23, HTRA2*), ubiquitin proteases (*USP9X*), lysosomal proteases (*AGA, PRCP*), a regulator of the inflammatory response and apoptosis (*CASP1*), and matrix metalloproteases (*MMP-14*, also known as *MT1-MMP*). Genes encoding proteases with significantly downregulated expression included aminopeptidase N (ANPEP) and kallikreins (KLK1, KLK7).

Having identified proteases that are overexpressed in VS, we next ascertained whether their overexpression has any downstream effects on the expression of other genes or gene networks. Ingenuity Pathway Analysis (IPA) was performed based on the gene expression data, and *MMP-14* was independently predicted as a significant upstream regulator of the signaling networks (Supplementary Figure S1B). MMPs play significant roles in nearly every hallmark of cancer and many members, including MMP-14, have been investigated as potential diagnostic and prognostic biomarkers. Other members of the MMP family, such as MMP-9, have also been studied in VS and shown to be correlated with VS growth. The role of MMP-14 in VS has not been investigated previously. We, therefore, selected MMP-14, the most abundantly differentially expressed MMP, for further analysis as a candidate VS biomarker.

### MMP-14 Expression in VS and Healthy Nerve Controls

We measured the level of MMP-14 in tumor secretions from primary VS cultures using enzyme-linked immunosorbent assay (ELISA). MMP-14 was found to be significantly elevated in VS but not in GAN conditioned media controls (Figure 1B). We next examined the mRNA transcript levels of a panel of MMPs (*MMP-2, 7, 9, 17*, and *19*) in addition to *MMP-14* in HEI-193 cells, an NF-2 derived transformed human VS cell line. We found that *MMP-14* is the most abundantly expressed enzyme relative to other MMP family members (Figure 1C). The difference in gene expression was much more dramatic in freshly derived primary VS cultures, where *MMP-14* mRNA was overexpressed by over 100-fold (Figure 1D).

**Figure 2:**
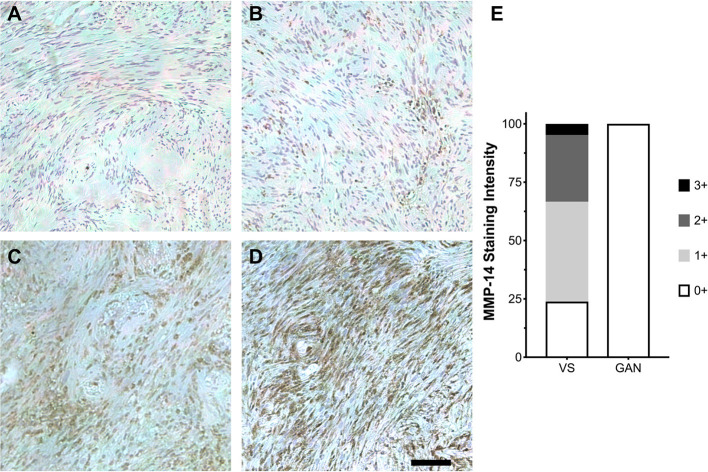
Expression of MMP-14 protein in human VS. **(A–D)** Immunohistochemical staining (IHC) of MMP-14 protein in archived VS specimens from patients with sporadic, unilateral VS. Staining is graded ranging from mild (0+, **A**) to mildly moderate (1+, **B**), moderate (2+, **C**), and intense (3+, **D**). Representative IHC from 10–12 histological sections per specimen is shown. A total of 21 different VS and 6 GAN specimens were analyzed. Scale bar, 100 μm. **(E)** Corresponding MMP-14 IHC staining scores for VS (*n* = 21) vs. GAN controls (*n* = 6).

Having established that MMP-14 is significantly overexpressed in both a schwannoma cell line and primary VS tumor culture, we next investigated the *in vivo* pattern of MMP-14 expression in formalin-fixed, paraffin-embedded tumor specimens from patients with VS as well as in GAN control sections. Within the tumor parenchyma, there were clusters of tumor cells that stained strongly positive for MMP-14. Globally, the overall expression of MMP-14 varied between individual tumors (Figure 2). A small fraction of the tumors had elevated expression in nearly all tumor cells (staining score 3+), whereas most specimens had at least mild to moderate expression of MMP-14. By contrast, no MMP-14 was detected in GAN specimens (Figure 2E; Supplementary Figure S2).

### Correlation of Plasma MMP-14 and Audiometric Data

**Figure 3:**
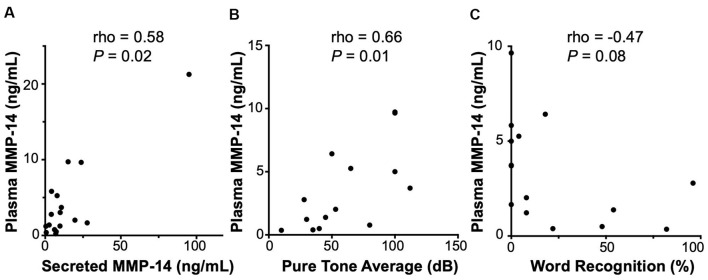
Correlation of MMP-14 with preoperative sensorineural hearing loss (SNHL) in VS patients. **(A)** Plasma MMP-14 levels positively and significantly correlate with tumor secreted MMP-14 from the same patients (*n* = 19). **(B)** MMP-14 plasma levels correlate positively and significantly with the degree of ipsilateral SNHL as measured by pure tone average (PTA; dB). **(C)** MMP-14 plasma levels correlate negatively with word recognition (WR) scores (%). *P-*values are shown and rho represents Spearman’s rank correlation coefficient.

A clinically useful biomarker should be readily sampled *via* a simple blood draw without the need for invasive procedures or biopsies. Therefore, we next set out to investigate the expression of circulating MMP-14 in the plasma of VS patients preoperatively and correlate it with annotated clinical and audiometric data. MMP-14 present in the peripheral blood correlated positively and significantly with the level of MMP-14 found in tumor secretions obtained from the same patients (rho = 0.58, *P* = 0.02, Figure 3A), suggesting that plasma MMP-14 levels may be used as a surrogate marker for tumor-secreted MMP-14.

We next examined the relationship between the level of MMP-14 and the degree of preoperative hearing loss. Patients from whom MMP-14 levels were obtained had varying degrees of ipsilateral SNHL, with PTA ranging from 8 to 100 dB and word recognition score (WRS) from 0 to 100%. MMP-14 levels were found to be ranging from 0.21 to 9.73 ng/ml (mean, 3.07 ng/ml). Plasma MMP-14 levels correlated positively and significantly with the subjects’ degree of hearing loss measured by PTA (rho = 0.66, *P* = 0.01, Figure 3B). The level of MMP-14 trended to correlate negatively with WRS (rho = −0.47, *P* = 0.08, Figure 3C), although this trend did not meet statistical significance.

### Establishing an Assay for MMP-14 Proteolytic Activity

**Figure 4:**
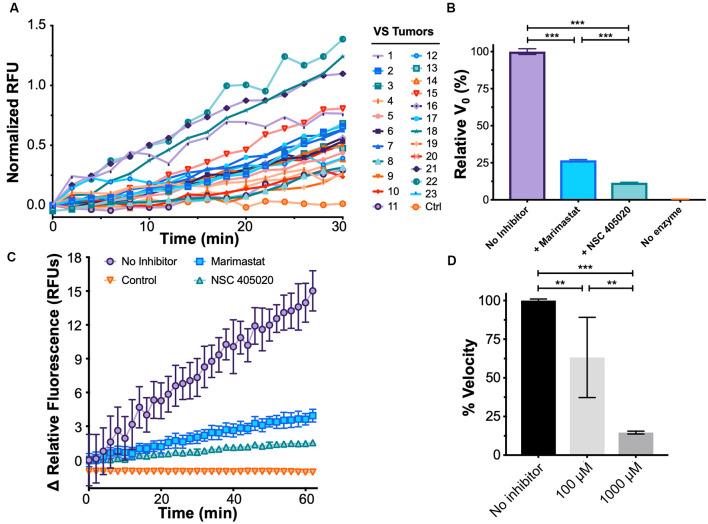
Development of a functional assay to sense plasma MMP-14 activity in VS. **(A)** Fluorescence de-quenching measurements of protease-mediated cleavage of MMP-14 specific fluorescent resonance energy transfer (FRET) substrate, using plasma samples obtained from VS patients. Controls indicate no added specimen. **(B)** Fluorescence de-quenching is MMP-14 specific and is inhibited by both Marimastat (blue), a broad-spectrum protease inhibitor, and NSC 405020 (green), an MMP-14 specific inhibitor. Controls (orange) indicate no added specimen. Error bars represent SEM from *n* = 6 independent experiments. **(C)** Relative fluorescence measurements of MMP-14 cleavage in the presence of protease inhibitors. Controls (orange) indicate no added specimen. Error bars represent SEM from *n* = 6 independent experiments. **(D)** Dose-dependent inhibition of MMP-14 activity by NSC 405020. Error bars represent SEM from four to six independent experiments. **P < 0.01, ***P < 0.001 by one-way ANOVA.

Protease activity biomarkers may outperform existing markers owing to its catalytic nature for signal amplification (Kwong et al., 2015). To this end, in addition to measuring MMP-14 levels in the patient’s plasma, we developed a fluorescence energy-based transfer (FRET)-based assay with a substrate probe specific for MMP-14 to determine its proteolytic activity in the blood (Packard et al., 2009). We found MMP-14 proteolytic activity strongly and positively correlated with the subject’s plasma concentration of MMP-14 (rho = 0.847, *P* < 0.001); as a negative control, no discernable MMP-14 activity was detected in conditioned media from GAN cultures (Figure 4A and Supplementary Figure S3A).

We next probed whether the proteolytic activity measured by FRET was indeed due to MMP-14 through a series of inhibition experiments. NSC 405020 is a small molecule that targets the hemopexin domain of MMP-14 and specifically inhibits its proteolytic activity (Remacle et al., 2012). Proteolysis was inhibited by both NSC 405020 and Marimastat, a broad-spectrum MMP inhibitor; but inhibition was more potent with the MMP-14 specific inhibitor (Figures 4B–D). Specifically, the initial velocity of FRET substrate cleavage, V_0_, was reduced by 75% with Marimastat and by over 85% with NSC 405020 in a dose-dependent fashion. Finally, substrate concentration dependence on cleavage velocity was confirmed by fitting cleavage velocity to the Michaelis–Menten equation, with a catalytic efficiency (*k*_cat_*/K_M_*) of approximately 2.89 × 10^6^ M^−1^^s^^−1^ (Supplementary Figure S3B). Taken together, these results suggest that the FRET assay can accurately measure MMP-14-specific proteolytic activity.

### MMP-14 Activity Correlates With the Extent of Surgical Resection in VS Patients

In our cohort of patients with VS, 11 (47.8%) underwent STR while 12 (52.2%) had either near- or gross-total resection. In the era of modern microsurgery where functional preservation of the facial nerve is becoming paramount, less-than-total removal is often advocated in the case of larger tumors or cases where the facial nerve may be at risk. As tumors enlarge, they can compress the facial nerve which results in splaying and thinning of the nerve, as well as increased adherence at the tumor-nerve interface (Gurgel et al., 2012). We next sought to identify factors associated with the incomplete extent of resection by comparing the GTR and STR cohorts. There were no statistically significant differences in factors such as age (49.8 vs. 55.5 years, *P* = 0.372), tumor volume (7.14 vs. 15.6 ml, *P* = 0.11), and tumor growth rate (1.14 vs. 1.97 ml/year, *P* = 0.63). Furthermore, postoperative facial nerve function, as assessed by the HB scale, was not significantly different between the two cohorts (2.07 vs. 2.17, *P* = 0.09).

**Figure 5:**
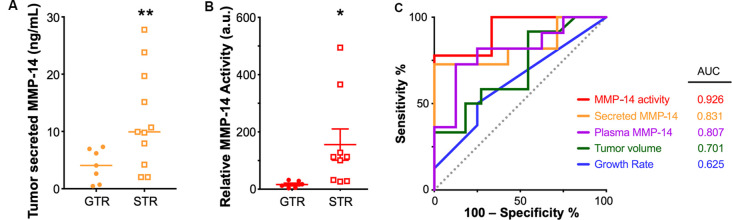
Elevated MMP-14 is associated with subtotal tumor resection (STR). **(A)** Comparison of the level of MMP-14 enzyme found in tumor secretions obtained from GTR (*n* = 7) vs. STR (*n* = 11) cohorts. Mean ± SEM is denoted by a horizontal line and error bars; two-tailed unpaired Student’s *t*-test, **P < 0.01. **(B)** Comparison of the total proteolytic activity of MMP-14, as determined by the plasma level of MMP-14 and its proteolytic activity, between patients who underwent GTR (*n* = 7) or STR (*n* = 9). Mean ± SEM is denoted by a horizontal line and error bars; two-tailed unpaired Student’s *t*-test, *P < 0.05. A.u., arbitrary units. **(C)** Receiver operating curves (ROC) and calculated area under curve (AUC) values for MMP-14 proteolytic activity, secreted MMP-14, plasma MMP-14, tumor volume, and growth rate. The gray line represents a predictor with no discriminatory power (AUC = 0.50).

By contrast, we found that sub-totally resected tumors secreted a much higher level of MMP-14 than completely resected tumors (9.93 vs. 4.06 ng/ml, *P* = 0.002, Figure 5A). Patients in the STR cohort also had significantly higher plasma levels of MMP-14 (3.73 vs. 1.00 ng/ml, *P* = 0.005) and increased MMP-14 proteolytic activity (*P* = 0.034, Figure 5B). In a multivariate logistic regression model, both plasma and tumor-secreted levels of MMP-14 remain independently associated with having a surgical outcome of STR (plasma MMP-14 OR = 5.43, 95% CI = 1.10–26.86, *P* = 0.038; secreted MMP-14 OR = 1.27, 95% CI = 1.05–1.55, *P* = 0.016), when patient age, gender and tumor size were taken into account (Supplementary Table S2). MMP-14 levels were not predictive of postoperative hearing preservation (Supplementary Table S3).

A comparison of the various diagnostic systems’ predictive powers demonstrated that MMP-14 activity (area under the curve based on receiver-operating curves, ROC-AUC = 0.926) exceeded that of either tumor growth rate (ROC-AUC = 0.625), tumor volume (ROC-AUC = 0.701), the level of plasma MMP-14 (ROC-AUC = 0.807) or tumor secreted MMP-14 (ROC-AUC = 0.831, Figure 5C). A possible explanation of this association between high MMP-14 activity and less-than-total tumor removal may be related to the degree of tumor adhesion to the facial nerve, as higher MMP-14 levels were associated with decreased amplitudes of intraoperative facial nerve stimulation (Supplementary Figure S4), which may lead to an STR outcome to avoid permanent facial nerve injury.

### Application of MMP-14 Leads to Neurite Loss in Cochlear Explants

**Figure 6:**
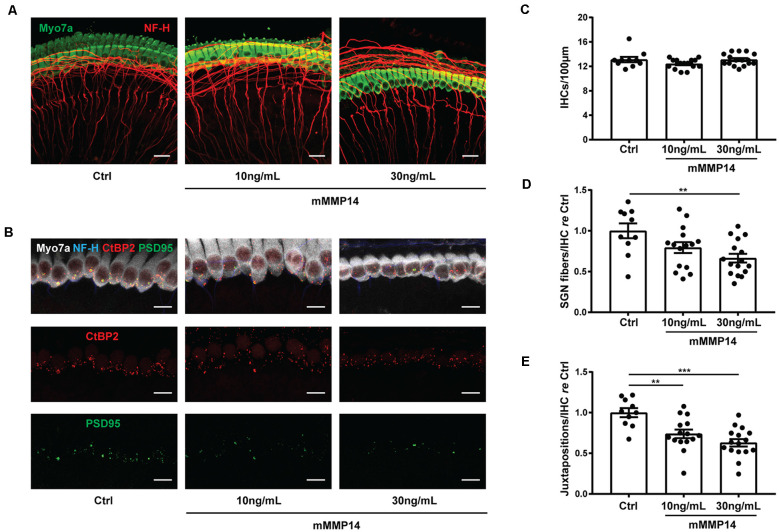
Application of MMP-14 to murine cochlear explants leads to loss of spiral ganglion neurites and cochlear synapses. **(A)** Representative images of cochlear explants receiving no treatment (Ctrl, *n* = 10 different explants), incubated with 10 ng/ml of murine MMP-14 (mMMP-14) for 72 h (*n* = 15 different explants), or with 30 ng/ml of murine MMP-14 for 72 h (*n* = 16 different explants). Myo7a (green) marks hair cells and NF-H (red) marks spiral ganglion neurites. Scale bar, 20 μm. **(B)** Representative images of untreated cochlear synapses (Ctrl, left column, *n* = 10 explants), synapses after incubation with 10 ng/ml of mMMP-14 (middle column, *n* = 15 explants), or 30 ng/ml of mMMP-14 (right column, *n* = 16 explants). CtBP2 (red) marks pre-synaptic ribbons and PSD95 (green) marks post-synaptic densities. Scale bar, 10 μm. **(C–E)** Quantification of the number of IHCs per 100 μm **(C)**, spiral ganglion neuron (SGN) fiber bundles per IHC measured 10 μm below the basolateral poles of hair cells, normalized to no-treatment controls **(D)**, and CtBP2-PSD95 juxtapositions per IHC normalized to no-treatment controls **(E)**. Error bars represent SEM. **P < 0.01, ***P < 0.001 by one-way ANOVA.

To understand if MMP-14 has the potential to directly cause SNHL in VS, we performed experiments in murine cochlear explant cultures to study the effects of MMP-14 on inner hair cells (IHCs), neurites of SGNs and synapses between them. Cell counts and morphology of MMP-14-treated explants were compared with those of age-matched, untreated controls. The amount of MMP-14 was chosen to be within the range of pathophysiologic levels measured in plasma of VS patients. After exposure to MMP-14 at either 10 or 30 ng/ml for 72 h, there was no significant change observed in the number of IHCs (*P* = 0.127, Figures 6A,C). However, a statistically significant loss in the number of nerve fiber bundles 10 μm below the basolateral pole of IHCs was observed at the higher MMP-14 concentration (1.00 ± 0.29 in control vs. 0.67 ± 0.21 in 30 ng/ml of MMP-14, *P* = 0.005, Figures 6A,D). Furthermore, the number of juxtapositions between CtBP2-positive presynaptic ribbons and PSD95-positive post-synaptic densities decreased significantly with increasing concentrations of MMP-14 (Figure 6B), from 1.00 ± 0.17 in the control cohort to 0.74 ± 0.20 after application of 10 ng/ml of MMP-14 (*P* = 0.004), decreasing further to 0.63 ± 0.18 after application of 30 ng/ml of MMP-14 (*P* < 0.001, Figure 6E). Taken together, our results suggest MMP-14 as a key mediator of SNHL in VS, *via* a toxic effect on SGN fibers and synapses in a dose-dependent fashion.

## Discussion

Although VSs are benign, their growth may still lead to significant morbidities due to the anatomic location and proximity to the facial and cochleovestibular nerves. Also, even non-growing tumors can cause progressive symptoms (Graamans et al., 2003). Therefore, a better understanding of the mechanisms of tumor growth and development of associated hearing loss may lead to novel biomarkers to aid in treatment decision making and potentially new therapies to augment current treatment modalities. Proteases are important enzymes central to numerous pathways in health and disease, including benign neoplasms such as VS (López-Otín and Bond, 2008). The abundance and activity of proteases such as MMPs are highly regulated by extensive post-translational modifications and a complex network of endogenous inhibitors. Therefore, precise activity-based protease sensors, rather than traditional transcriptomic or proteomic measurements, are needed to gain a better understanding of the disease process and to predict treatment outcomes. To this end, we developed a new FRET-based assay to detect plasma MMP-14 activity in VS patients. At a fundamental level, this represents a new paradigm in VS biomarker discovery, where the dynamic activity of the biomarker may provide important insights.

Here, the *in silico* identification of aberrantly expressed MMP-14 in human VS using pooled human transcriptomic data, combined with functional validation in primary VS cultures and mechanistic studies in cochlear explant models, together establishes a powerful platform for the discovery and validation of novel biomarkers for VS. Using this platform, we demonstrate that MMP-14 protease activity can lead to oto- and neurotoxicity seen in VS patients with SNHL, and elevated MMP-14 is observed in patients with STRs. Our study is novel for several reasons: (1) we discovered MMP-14, a member of the membrane-type MMP family with previously unknown roles in VS, that was significantly upregulated in VS; (2) using a new functional assay of MMP-14 proteolytic activity, we found significant differences between patients who underwent GTR compared to those with STR; (3) we identified an expanded set of protease candidate markers aberrantly expressed in primary VS tumor cultures; and (4) we provided the first mechanistic demonstration that MMP-14 leads to cochlear damage in a cochlear explant model.

Members of the MMP family, such as MMP-2 and MMP-9, have been implicated in promoting the “angiogenic switch” and neovascularization in VS. A series from Moon et al. (2007) found that cystic VS was more adherent to the facial nerve than solid tumors, and tumor cells adjacent to the cyst cavity showed increased MMP-2 expression. Although the exact mechanism of peritumoral adhesion was not investigated, the authors postulated that increased proteolysis of the tumor matrix may affect the tumor-nerve barrier (Moon et al., 2007). Another study examined the correlation between MMP-2 and MMP-9 expression with clinical features in patients with sporadic, non-cystic VS (Møller et al., 2010). MMP-2 was found in tumor homogenates by ELISA and its expression was localized to the extracellular matrix. MMP-9 was found in tumor cells and correlated with growth rate, possibly in a concerted fashion with VEGF signaling (Cayé-Thomasen et al., 2003). However, both studies only assessed a narrow set of MMPs in the post-operative setting; neither examined audiometric outcomes nor offered mechanistic insight into VS-associated SNHL. Interestingly, MMP-14 has been proposed as an activator of latent MMP-2 in conjunction with tissue inhibitors of metalloproteinase 2 (TIMP-2) for degradation of extracellular matrix and promotion of cancer invasion and metastasis (Nishida et al., 2008; Sato and Takino, 2010). Nevertheless, MMP-2 was not one of the proteases with dysregulated expression from our meta-analysis of the genome-wide transcriptional microarray data, and MMP-2 mRNA levels were not significantly upregulated in either human VS cultures or schwannoma cells *in vitro*. Together, our data motivate further evaluation of the roles other MMPs, in particular MMP-2 and MMP-9, play in interacting with MMP-14 to promote VS growth and development of associated hearing loss in larger datasets.

Activity-based analysis of protease activity ensures that MMP-14 is functional and therefore plays a causal role in VS progression. Previous studies implicating MMP-2 and MMP-9 in VS only measured their abundance levels and did not provide functional activity measurements (Moon et al., 2007; Møller et al., 2010). Protease activity-based probes, such as the FRET-based assay developed here, have the potential for multiplexed simultaneous assays and exceed the sensitivity of traditional biomarkers through signal amplification (Hori and Gambhir, 2011; Kwong et al., 2013). Many new techniques to assay protease activity for cancer detection and prediction are under development (Sanman and Bogyo, 2014). Therefore, given these new areas of exciting development, further optimization of the assay could have significant diagnostic and prognostic implications for patients with VS.

Historically, GTR was often the standard treatment for nearly all VS at the expense of normal facial nerve function, regardless of tumor size or patient comorbidity. As VS grows, the tumor-arachnoid mater plane is gradually lost and the facial nerve can become splayed and thinned, making complete tumor resection difficult (Comey et al., 1995; Bloch et al., 2004). The likelihood of complete tumor removal can be affected by a combination of cellular proliferation, tumor vascularity, inflammation in the tumor microenvironment, presence of cystic degeneration, and the surgeon’s experience. More recently, there has been a shift in VS management towards STR to maximize the preservation of neurological function and quality of life (Ramsden, 1995; Babu et al., 2013). Today, approximately 50% of VS patients undergo primary microsurgery and 18% of those undergo STR in the United States (Carlson et al., 2015a). However, STR has been associated with a higher likelihood of tumor recurrence ranging from 22 to 55% (Seol et al., 2006; Carlson et al., 2012; Monfared et al., 2016), which would require more frequent long-term follow-ups, and further intervention should tumor recurrence occur. Nonetheless, there are currently no molecular biomarkers that could accurately inform the degree of SNHL or the extent of surgical resection, two important features that undoubtedly impact the disease prognosis and in turn, the patient’s long-term quality of life.

In our study, neither tumor size nor the absolute growth rate was associated with the likelihood of complete tumor removal; this was likely a reflection of the heterogeneous tumor biology that is more complex than the aggregate tumor size or growth rate. By contrast, the proteolytic activity of MMP-14 was significantly different between GTR vs. STR groups, and the tumor-secreted and plasma levels of MMP-14 was found to be independent predictors of less than total tumor removal in multivariate regression analysis. Mechanistically, elevated MMP-14 activity is likely accompanied by increased extracellular matrix remodeling and vascular changes surrounding the tumor, which results in an upregulation of the inflammatory reaction that ultimately increases the adhesion between the tumor capsule and surrounding structures such as the facial and cochlear nerves.

Cross-referencing a database of proteolytic enzymes and the pooled transcriptomic dataset of VS led to the identification of multiple protease markers that are upregulated in VS. The identification of CASP1 and HTRA2, a mitochondrial serine protease mediating apoptosis in Parkinson’s disease, also highlights the important role of caspase-dependent apoptosis in merlin-deficient Schwann cells in an *in vitro* model of NF2 (Fuse et al., 2017). Moreover, given the possible therapeutic efficacy of NSAIDs and curcumin against schwannomas, it is not surprising that PRCP, a mediator of inflammation and upstream regulator of angiotensin and bradykinin, was identified (Dilwali et al., 2015a). Several additional proteases have been implicated in NF1 and malignant peripheral nerve sheath tumors (MPNSTs), two neuro-cutaneous syndromes that share features with NF2. For example, USP9X is a deubiquitinating enzyme overexpressed in malignant nervous system tumors and plays a critical role in MPNST cell survival (Bianchetti et al., 2018). Interestingly, emerging data in the literature suggest kallikreins may be directly involved in neoplastic progression by negatively regulating tumor growth in breast and colon cancers (Borgoño and Diamandis, 2004). Both KLK1 and KLK7 were found to be downregulated in our analysis, which motivates future work in investigating the functional and clinical implications of kallikreins in VS.

Similar to other ototoxic molecules such as TNFα, where tumor secretions containing high levels of TNFα led to damage in IHCs and nerve fibers in cochlear explants and loss of synapses *in vivo* (Dilwali et al., 2015b; Katsumi et al., 2020), application of murine MMP-14 at nanomolar levels onto cochlear explants induced significant damage to nerve fiber and synaptic juxtapositions at the basal pole of IHCs, thereby validating the oto- and neurotoxic role of MMP-14. This may provide mechanistic insight into the common clinical observation that word recognition in VS patients is typically disproportionally worse than pure tone audiometric thresholds because word recognition is markedly influenced by the integrity of the SGNs and their synapses on hair cells. In our cohort, we identified a significant correlation between plasma MMP-14 level and the degree of SNHL in patients with VS as assessed by PTA; a similar trend was observed between MMP-14 and WR scores. Our study is the first to identify a putative ototoxic role of MMPs in the context of VS. Future studies warrant further investigations into whether antibody- or small-molecule mediated MMP-14 inactivation could in turn prevent cochlear explant damage, and possibly the use of clinical MMP inhibitors to alleviate SNHL.

This study has several limitations. First, the small sample size hindered our ability to broadly generalize our findings. Second, due to the limited clinical data available for certain historic patients, the complete natural history of tumor growth and long-term audiometric data were not available in some cases. Third, while the FRET assay to assess protease function was validated in plasma samples, the presence of other protease enzymes and regulators of protease activity could influence the assay’s specificity.

Looking forward, to move this technology towards potential clinical translation, the results from this study should be validated in a larger cohort of surgical patients. Prospective evaluation by deploying MMP-14 functional assays preoperatively may inform the surgeon of the likelihood of successful tumor removal, thereby providing prognostic information to aid in patient counseling. Examination of MMP-14 levels in the serum of patients with post-radiated tumors or after treatment of primary VS cultures with ionizing radiation may provide insight into radiation-mediated changes in tumor biology. Encouragingly, mathematical modeling based on the activity of other MMPs demonstrated successful detection of millimeter-sized tumors (Kwong et al., 2015). A future area of investigation could include serial assessments of plasma MMP-14 activity in patients with small but growing VS, to quantify the sensitivity of the protease assay relative to traditional blood-based or imaging markers. In addition to plasma and tumor secretions, the perilymph fluid is enriched in proteins secreted by cells of the inner ear and VS and represents another promising source of biomarker discovery (Lysaght et al., 2011). As more targets emerge from integrative analyses of schwannoma genomes, our current platform can be adapted to investigate other proteases as functional biomarkers or therapeutic targets. In the future, MMP-14 activity-based sensors may be deployed intraoperatively to identify potential areas of high peritumoral adhesion to assist in surgical removal.

## Conclusion

In summary, we report the identification of multiple protease enzymes aberrantly expressed in VSs. Focusing on MMP-14, a member of the membrane-type MMP family with previously unknown roles in VS tumorigenesis or SNHL, we optimized a new functional assay to measure its proteolytic activity in plasma samples. MMP-14 levels correlated with preoperative hearing thresholds. In patients who underwent subtotal tumor removal, tumor secreted MMP-14 and plasma MMP-14 activity were both significantly elevated compared to patients with complete tumor resection. Mechanistically, we demonstrated direct neuronal damage, including loss of neurites and cochlear synapses, due to MMP-14 in a cochlear explant model. Taken together, our findings expand the understanding of the role of proteases in VS and identify other candidate proteases from the largest pooled transcriptomic analysis to date that, when validated, could further enhance the diagnosis and personalized treatment of VS.

## Data Availability Statement

All datasets generated for this study are included in the article/Supplementary Material.

## Ethics Statement

The studies involving human participants were reviewed and approved by Human Studies Committee of Massachusetts General Hospital and Massachusetts Eye and Ear. The patients/participants provided their written informed consent to participate in this study. The animal study was reviewed and approved by Human Studies Committee of Massachusetts General Hospital and Massachusetts Eye and Ear.

## Author Contributions

YR and KS designed research. YR performed experiments. HH performed cochlear explant studies. JS and LL provided VS and Schwann cell cultures. YR, HH, and KS analyzed data. YR and KS wrote the manuscript. DW helped with specimen collection. YR, HH, JS, LL, DW, and KS critically edited the manuscript.

## Conflict of Interest

The authors declare that the research was conducted in the absence of any commercial or financial relationships that could be construed as a potential conflict of interest.
