# Supplementary material for: MMP-14 (MT1-MMP) Is a Biomarker of Surgical Outcome and a Potential Mediator of Hearing Loss in Patients With Vestibular Schwannomas
Source: Front Cell Neurosci. 2020 Jul 28;14:191. doi: 10.3389/fncel.2020.00191 (PMC7424165; doi:10.3389/fncel.2020.00191)
Supplement: Supplementary file 1 [file Table_1.docx]

| **Description** | **Gene**  **Name** | **Bonferroni** | **Weighted fold change** |
| --- | --- | --- | --- |
| A disintegrin and metalloproteinase 23 | ADAM23 | 0.0017 | 4.89 |
| Umbilical vein proteinase | PRSS23 | 0.0001 | 4.28 |
| Caspase-1 | CASP1 | 0.0111 | 3.48 |
| Glycosylasparaginase | AGA | 0.0062 | 3.24 |
| USP9X | USP9X | 0.0296 | 3.01 |
| Beta lactamase | LACTB | 0.0265 | 2.92 |
| Plasma Glu-carboxypeptidase | CPQ | 0.0314 | 2.74 |
| HTRA2 | HTRA2 | 0.0001 | 2.70 |
| Membrane type matrix metalloprotease 1 | MMP14 | 0.0111 | 2.46 |
| Lysosomal Pro-X C-peptidase | PRCP | 0.0188 | 2.32 |
| Mesotrypsin | PRSS3 | 0.0152 | -1.67 |
| Family with sequence similarity 108, member C1 | ABHD17C | 0.0382 | -2.71 |
| Gln-fructose-6-P transamidase 2 | GFPT2 | 0.0221 | -2.94 |
| Kallikrein hK7 | KLK7 | 0.0035 | -3.03 |
| Kallikrein hK1 | KLK1 | 0.0003 | -3.31 |
| Aminopeptidase N | ANPEP | 0.0487 | -3.33 |

**Supplementary Table 1.** List of candidate proteases that are found to be significantly differentially expressed from combining the Human Degradome and meta-analysis of vestibular schwannoma gene expression.
