# Supplementary material for: MMP-14 (MT1-MMP) Is a Biomarker of Surgical Outcome and a Potential Mediator of Hearing Loss in Patients With Vestibular Schwannomas
Source: Front Cell Neurosci. 2020 Jul 28;14:191. doi: 10.3389/fncel.2020.00191 (PMC7424165; doi:10.3389/fncel.2020.00191)
Supplement: Supplementary file 2 [file Table_2.docx]

| **Variables** | **Odds Ratio** | **95% Confidence Interval** | ***P* value** |
| --- | --- | --- | --- |
| Age, years | 1.036 | 0.955 – 1.124 | 0.395 |
| Gender, vs. female | 13.092 | 0.384 – 446.59 | 0.153 |
| Tumor size, mL | 1.502 | 0.967 – 2.332 | 0.070 |
| Plasma MMP-14, ng/mL | 5.429 | 1.098 – 26.855 | 0.038 |

| **Variables** | **Odds Ratio** | **95% Confidence Interval** | ***P* value** |
| --- | --- | --- | --- |
| Age, years | 1.023 | 0.965 – 1.086 | 0.848 |
| Gender, vs. female | 3.117 | 0.330 – 29.44 | 0.321 |
| Secreted MMP-14, ng/mL | 1.271 | 1.045 – 1.546 | 0.016 |

**Supplementary Table 2.** Results of multivariate regression analysis of prognostic factors for surgical outcome of subtotal resection (STR).
