# Supplementary material for: MMP-14 (MT1-MMP) Is a Biomarker of Surgical Outcome and a Potential Mediator of Hearing Loss in Patients With Vestibular Schwannomas
Source: Front Cell Neurosci. 2020 Jul 28;14:191. doi: 10.3389/fncel.2020.00191 (PMC7424165; doi:10.3389/fncel.2020.00191)
Supplement: Supplementary file 3 [file Table_3.docx]

| **Variables** | **Odds Ratio** | **95% Confidence Interval** | ***P* value** |
| --- | --- | --- | --- |
| Age, years | 1.001 | 0.919 – 1.091 | 0.979 |
| Gender, vs. female | 0.131 | 0.009 – 1.944 | 0.140 |
| Tumor size, mL | 0.922 | 0.721 – 1.179 | 0.517 |
| Plasma MMP-14, ng/mL | 1.108 | 0.629 –1.951 | 0.723 |

| **Variables** | **Odds Ratio** | **95% Confidence Interval** | ***P* value** |
| --- | --- | --- | --- |
| Age, years | 0.983 | 0.905 – 1.068 | 0.688 |
| Gender, vs. female | 0.095 | 0.008 – 1.110 | 0.061 |
| Secreted MMP-14, ng/mL | 0.611 | 0.811 – 1.131 | 0.611 |

**Supplementary Table 3.** Results of multivariate regression analysis of prognostic factors for hearing preservation, defined as having a pure tone average (PTA) of less than 50 dB or a WRS of greater than 50% on postoperative audiogram.
