# Supplementary material for: MMP-14 (MT1-MMP) Is a Biomarker of Surgical Outcome and a Potential Mediator of Hearing Loss in Patients With Vestibular Schwannomas
Source: Front Cell Neurosci. 2020 Jul 28;14:191. doi: 10.3389/fncel.2020.00191 (PMC7424165; doi:10.3389/fncel.2020.00191)
Supplement: Supplementary file 4 [file Data_Sheet_1.PDF]

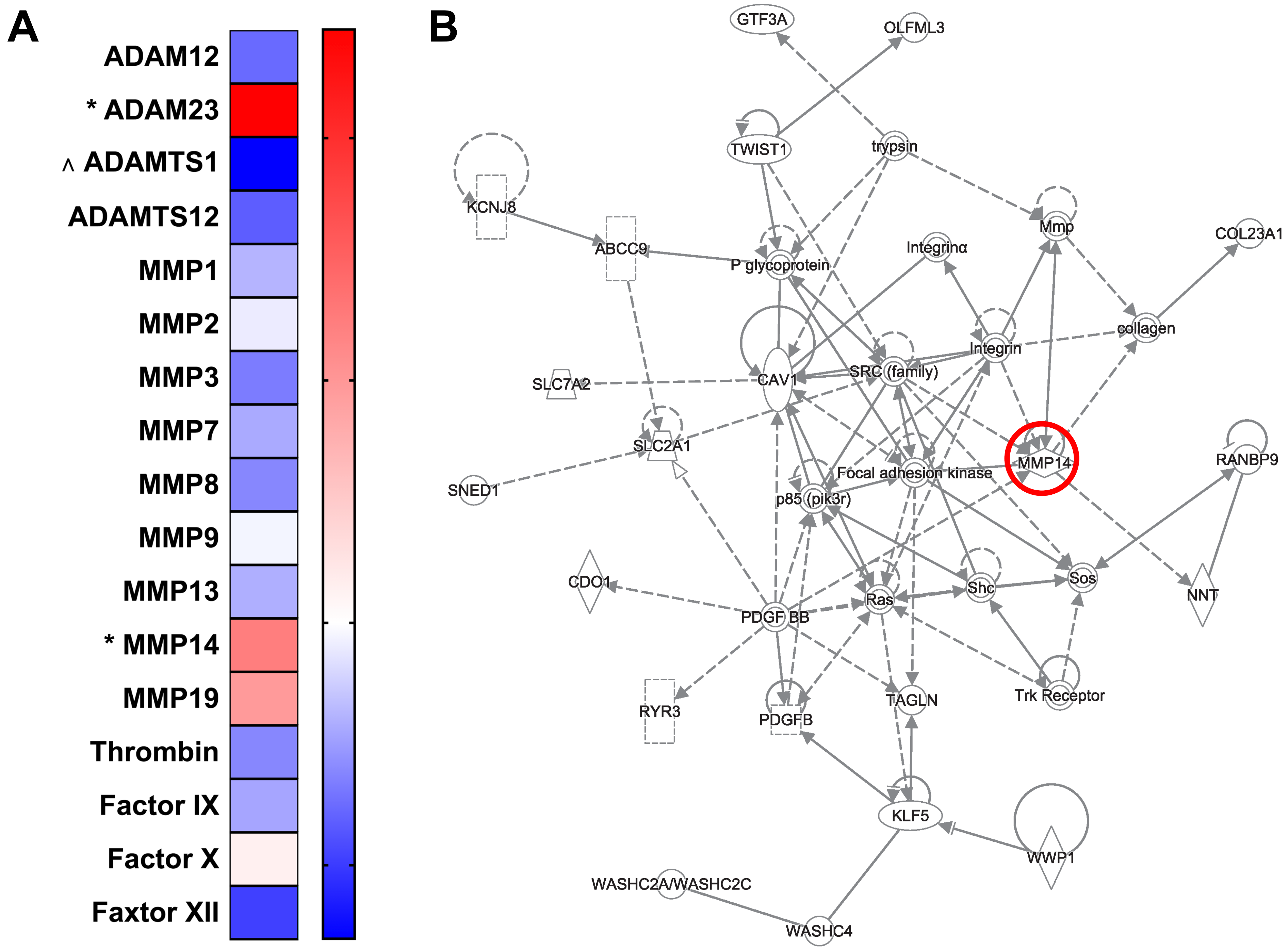

A
ADAM12
* ADAM23
^ ADAMTS1
ADAMTS12
MMP1
MMP2
MMP3
MMP7
MMP8
MMP9
MMP13
* MMP14
MMP19
Thrombin
Factor IX
Factor X
Faxtor XII
B
GTF3A
OLFML3
TWIST1
trypsin
KCNJ8
ABCC9
P glycoprotein
Integrinα
Mmp
COL23A1
collagen
Integrin
SRC (family)
CAV1
SLC7A2
SLC2A1
SNED1
MMP14
Focal adhesion kinase
p85 (pik3r)
RANBP9
CDO1
Shc
Sos
Ras
NNT
PDGF BB
RYR3
PDGFB
TAGLN
Trk Receptor
KLF5
WWP1
WASHC2A/WASHC2C
WASHC4

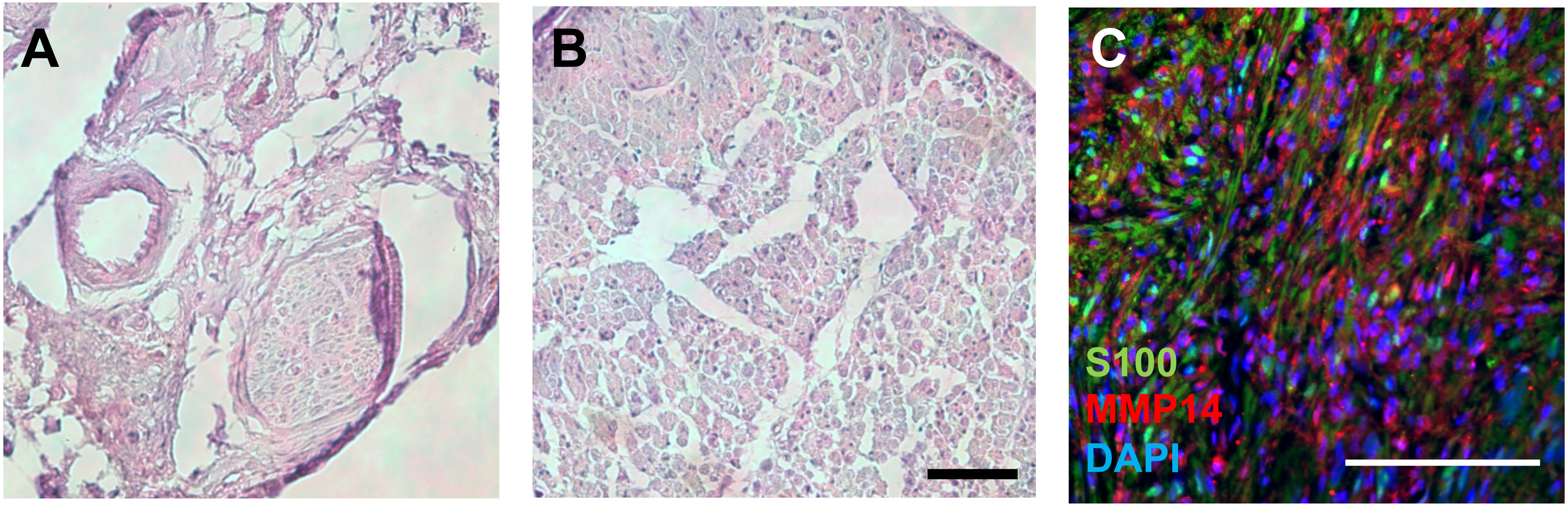
A
B
C
S100
MMP14
DAPI

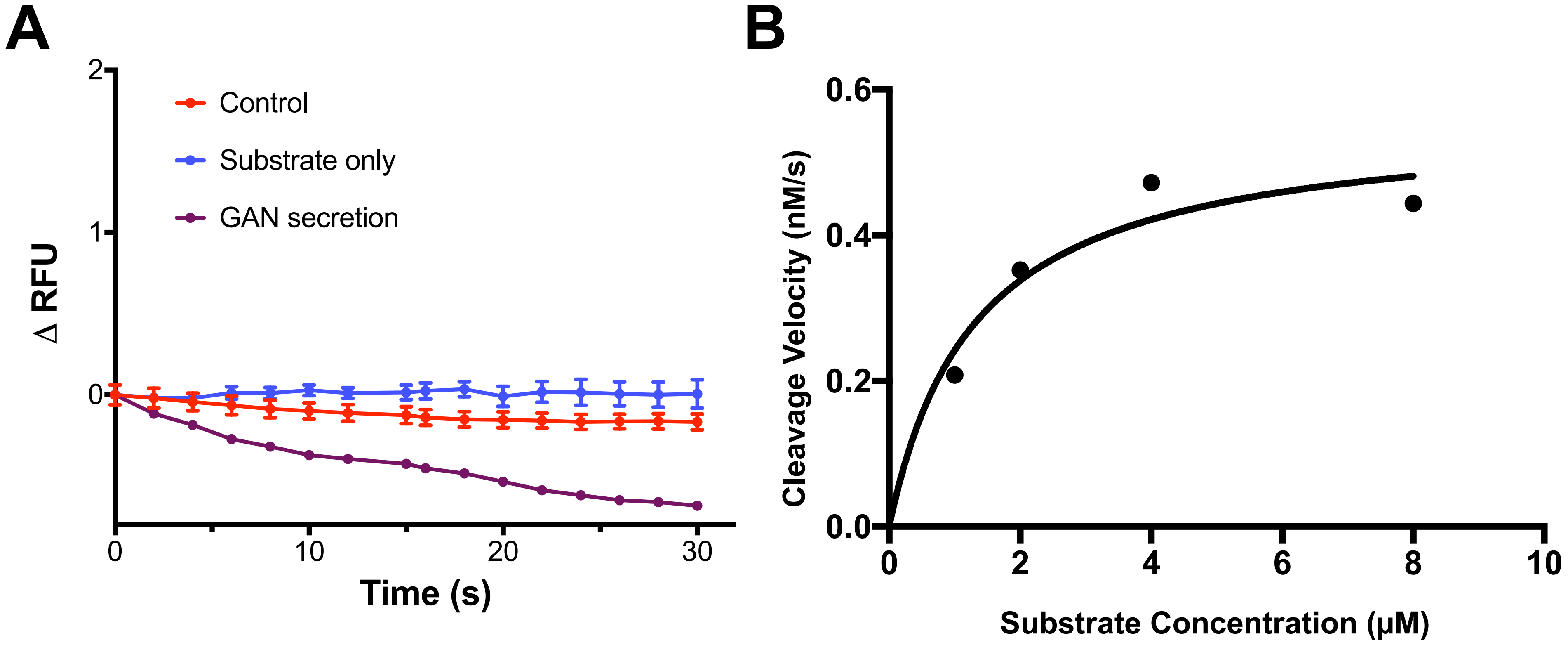
A
B
2
1
0
Δ RFU
Control
Substrate only
GAN secretion
0
10
20
30
Time (s)
0.6
0.4
0.2
0.0
Cleavage Velocity (nM/s)
0
2
4
6
8
10
Substrate Concentration (μM)

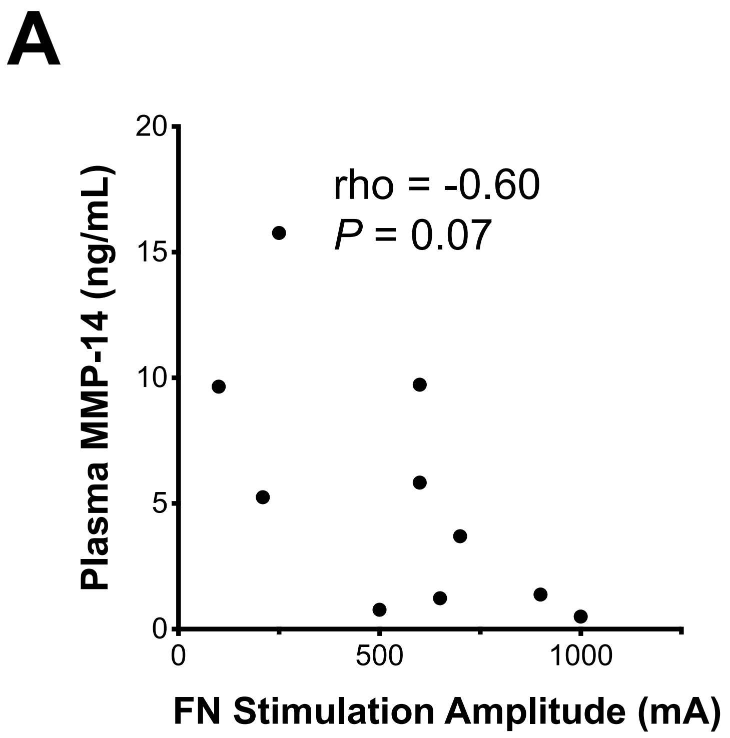
A
rho = -0.60
P = 0.07
Plasma MMP-14 (ng/mL)
0
5
10
15
20
0
500
1000
FN Stimulation Amplitude (mA)
B
Plasma MMP-14 (ng/mL)
0
5
10
15
20
25
**
Gross total
Subtotal
Extent of Resection
