# Supplementary material for: MMP-14 (MT1-MMP) Is a Biomarker of Surgical Outcome and a Potential Mediator of Hearing Loss in Patients With Vestibular Schwannomas
Source: Front Cell Neurosci. 2020 Jul 28;14:191. doi: 10.3389/fncel.2020.00191 (PMC7424165; doi:10.3389/fncel.2020.00191)
Supplement: Supplementary file 5 [file Data_Sheet_2.DOCX]

**TITLE:** MMP-14 (MT1-MMP) is a biomarker of surgical outcome and potential mediator of hearing loss in patients with vestibular schwannomas

**AUTHORS:** Yin Ren, Hiroshi Hyakusoku, Jessica E. Sagers, Lukas D. Landegger, D. Bradley Welling and Konstantina M. Stankovic

**Supplementary Information**

- Figure caption for Supplementary Figures 1 – 4

**SUPPLEMENTARY FIGURE CAPTIONS**

**Supplementary Figure 1. MMP-14 is significantly upregulated in VS. A.** Heat map of gene expression of clinically relevant proteases in VS. Blue indicates low expression, and red indicates high expression. **B.** Ingenuity Pathway Analysis (IPA) of genes differentially expressed in patients with VS versus controls highlights MMP-14 (circled in red) as a nodal molecule in one of the top signaling pathways. Solid lines indicate direction connections. Dashed lines indicate indirect connections.

**Supplementary Figure 2. Expression of MMP-14 protein in VS and great auricular nerve. A-B.** Immunohistochemical (IHC) staining of MMP-14 in human great auricular nerve (GAN) sections revealed no specific staining. Scale bar, 100 µm. Representative sections based on analysis of 6 different GAN specimens. **C.** Immunofluorescence staining of a neural crest marker (S100, green) and MMP-14 (red) in VS. Cell nuclei are counterstained with DAPI (blue). Scale bar, 100 µm. A representative section based on analysis of 21 different VS specimens was shown.

**Supplementary Figure 3. Development of a functional assay to sense serum MMP14 activity. A.** Control secretions from great auricular nerves (GAN) do not cleave the FRET substrate. Error bars represent SEM. N = 3 independent experiments. **B.** Michaelis-Menten analysis of MMP-14 cleavage of FRET substrate.

**Supplementary Figure 4. Association of serum MMP-14 and clinical factors in VS patients. A.** Serum MMP-14 is indirectly associated with the amplitude of intraoperative facial nerve stimulation (n = 10), which trended towards statistical significance. *P-*values are shown and rho represents Spearman’s rank correlation coefficient. **B.** Comparison of serum level of MMP-14 between GTR versus STR patient cohorts. Mean +/- SEM is denoted by a horizontal line and error bars; two-tailed Student’s *t*-test, ***P* < 0.01.
